# Public transportation and transmission of viral respiratory disease: Evidence from influenza deaths in 121 cities in the United States

**DOI:** 10.1371/journal.pone.0242990

**Published:** 2020-12-01

**Authors:** Renata E. Howland, Nicholas R. Cowan, Scarlett S. Wang, Mitchell L. Moss, Sherry Glied

**Affiliations:** 1 Robert F. Wagner Graduate School of Public Service, New York University, New York, NY, United States of America; 2 Rudin Center for Transportation Policy & Management, New York University, New York, NY, United States of America; Chinese University of Hong Kong, HONG KONG

## Abstract

One important concern around the spread of respiratory infectious diseases has been the contribution of public transportation, a space where people are in close contact with one another and with high-use surfaces. While disease clearly spreads along transportation routes, there is limited evidence about whether public transportation use itself is associated with the overall prevalence of contagious respiratory illnesses at the local level. We examine the extent of the association between public transportation and influenza mortality, a proxy for disease prevalence, using city-level data on influenza and pneumonia mortality and public transit use from 121 large cities in the United States (US) between 2006 and 2015. We find no evidence of a positive relationship between city-level transit ridership and influenza/pneumonia mortality rates, suggesting that population level rates of transit use are not a singularly important factor in the transmission of influenza.

## Introduction

Concern about the role of public transit use in the spread of contagious respiratory diseases is long-standing, widespread, and very timely. The literature on the epidemiology of influenza, in particular, dates back to the early 1900s and has historically demonstrated that mortality from season influenza and pneumonia is concentrated among older adults and among those with pre-existing health conditions [1, 2]. It also points to sociodemographic characteristics, including income, race and ethnicity, as possible risk factors potentially because these characteristics are correlated with underlying health conditions or with increases in the probability of exposure to disease [3].

One source of such increased exposure is hypothesized to be the use of public transit [4]. Public perceptions of transit also reflect this. In an analysis of the severe acute respiratory syndrome (SARS) outbreak in 2003, Sadique et a. (2007) found that 75% of respondents reported they would avoid public transportation due to concern that it would place them at high risk for exposure [5]. During the COVID-19 pandemic, substantial reductions in public transit have in fact been reported in many major cities [6]. For example, even prior to stay-at-home orders, ridership in New York City subways was down 50% as of March 15, 2020 compared to the same day the previous year [7]. Despite these concerns, the evidence is mixed about whether exposure to public transit is any more dangerous than interactions that occur in schools, workplaces, businesses, religious gatherings, sporting events, and neighborhoods and whether the use of public transit increases the rate of community transmission of influenza, even among those who do not themselves use transit.

Based on our review of the literature, few studies have directly examined the potential role of public transit use in the spread of contagious respiratory diseases. In a study of influenza-like illness and time spent in the London Underground, Goscé and Johansson (2018) found that incidence was higher in areas where commutes to Central London involved more contact with other riders; however, the authors included only one year of data and did not directly measure the share of people in each borough who ride the underground; instead, they simply controlled for car ownership [8]. In a small case-control study following the 2008–2009 flu season, Troko et al. (2011) found that recent bus or tram use was associated with a higher risk of consulting a general practitioner for acute respiratory infection; however, this finding was attenuated once the authors controlled for habitual public transit use [9]. By contrast, Adler et al. (2014) found no correlation between influenza rates and transit commuting patterns across the United Kingdom based on responses to an internet-based survey in an open community cohort [10]. Moreover, in an agent-based simulation in New York City, Cooley et al. (2011) found that only 4.4% of the 2.6 million simulated infections would occur on the subway, suggesting transit-related interventions would be ineffective in slowing transmission [11].

While transportation of any type is clearly associated with the spread of disease from one geographic area to another, the role that public transportation plays in the transmission of disease within a local area is complex and difficult to study. One way to examine the full impact of mass transit on the spread of influenza is, therefore, to examine data aggregated to the city level, exploiting the considerable variation in the use of public transit across American cities. Therefore, we draw from a decade’s worth of city-level influenza/pneumonia mortality and census-based transit-use data to examine the relationship between community level use of public transportation and mortality from influenza/pneumonia. It is important to note that we are not examining whether influenza rates are higher among those who take public transit relative to those who do not; rather, we are assessing whether community transmission of severe influenza, including to those who never take transit, is greater in areas where transit use is higher.

## Materials and methods

The Centers for Disease Control and Prevention (CDC) report aggregate data on deaths where influenza and pneumonia is the underlying or contributing caused listed on death certificates (since these can be difficult to distinguish, see [2]). We downloaded and summarized CDC data by city and flu year (July 1, 2006 –June 30, 2016) from 2006 to 2015 for 121 cities in the US, most of which have populations over 100,000 [12]. To determine the extent of public transit ridership by city, we downloaded 5-year average American Community Survey (ACS) estimates for the periods 2006–2010 and 2011–2015 [13]. Our primary measure of transit ridership was based on responses to the question, “How did the person usually get to work LAST WEEK?”, summarized at the city level as the percentage of individuals over 16 years of age who take public transportation to work. In sensitivity analyses, we used the percentage of the population who commute 60 minutes or more on public transit as the measure of transit use. We also used the ACS to obtain city-level population estimates, age distributions (percent of the population <5, 5–24, 25–44, 45–64, and 65+), racial distribution, poverty, median income, educational attainment, food stamp recipiency, labor force participation, and unemployment rates.

Datasets were merged based on city, state name, and year to construct a city-year data set, excluding city-years where no data were present (n = 1201). We used negative binomial models to estimate influenza/pneumonia death counts (summed together), with an offset term for the population and robust standard errors clustered on city. The baseline model included the percent of residents using public transit to commute to work, the age distribution (percent of the population <5, 25–44, 45–64, and 65+), and the flu year modeled as a set of dummy variables. The adjusted model added census division areas (of which there are nine) to control for climate-related factors, and city-level percentages of bachelor’s degree or higher, non-white, below poverty, unemployed, and male. To test the robustness of the model, we repeated the analysis for each individual year and used linear and Poisson distributions in place of the negative binomial specification. Because transit use is highly correlated with population density, and both factors might be expected to contribute to the spread of influenza, we also conducted a sensitivity analysis controlling for population density.

Since cities may differ in their health care infrastructures, we conducted sensitivity analyses with two indicators designed to capture the quality of and access to the healthcare system. First, we obtained influenza vaccination rates for adults 65 years old and older from the CDC’s Behavioral Risk Factor Surveillance System for approximately 90 overlapping metropolitan statistical areas [14]. Vaccination rates provide direct protection against influenza and are also a measure of overall health care quality. Second, we obtained all-cause mortality at the city level from the CDC [12].

To examine city-specific effects of changes in transit, we ran the analysis for the full period using fixed effects for each city, identifying the relationship between changes in transit use and changes in flu across the full period. Because there was very little change in transit use among cities over this period, we also identified the 10% of cities with the largest absolute increase and the 10% of cities with the largest absolute decrease in transit use between the two time periods (2006–2010 and 2011–2015) and plotted the association between the average death rate in the first three years of the data (2006–2008) and the last three years of data (2013–2015) to determine whether there was an appreciable change in the death rate. To counter the fact that pneumonia cases make up a larger portion of deaths in our rate and that these deaths may be driven by hospital rather than community acquired infection, we created a ratio of deaths from October 1 –March 30 and from April 1 –September 30. Since flu deaths primarily occur in the fall and winter, whereas pneumonia deaths occur all year round, this ratio represents a proxy for flu vs. pneumonia deaths in a given city and year [15]. We then used this ratio in a linear regression to test its relationship with percent transit, adjusting for all the same variables from the negative binomial model. As a final sensitivity check against outliers, we conducted a Monte Carlo study, repeating the adjusted negative binomial model 100 times, and randomly selecting 75% of cities each time.

Throughout, we report the incidence rate ratio (IRR) for transit ridership and its associated confidence intervals. Coefficients and standard errors for the full model are provided in the S1 Appendix. All analyses were conducted in SAS 9.4 (Cary, North Carolina). Aggregate, publicly-available data were used for this analysis, therefore this study did not require human subjects research review.

## Results and discussion

**Fig 1:**
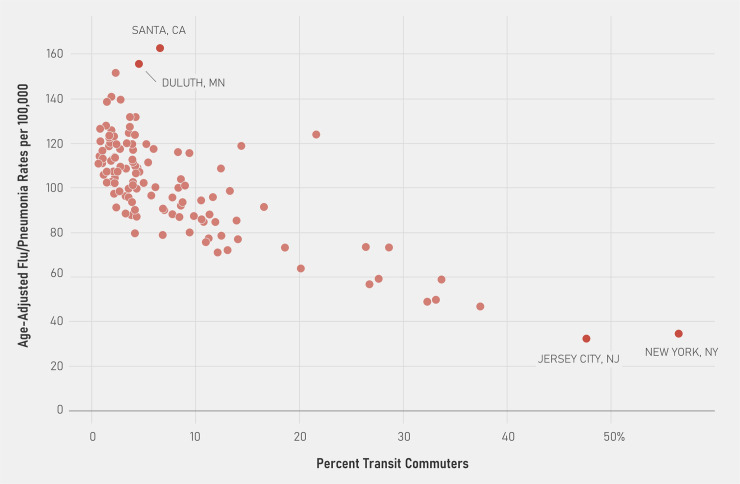
Population-adjusted influenza /pneumonia death rates compared with public transit ridership in 121 major cities in the United States, 2015. Source: CDC Weekly Influenza/Pneumonia Deaths from 2015 linked with ACS transit data for 2010–2015 by city and adjusted by the city age-distribution in a negative binomial regression.

In 2015, influenza/pneumonia death rates ranged from 0.65 per 100,000 in Springfield, MA to 329 per 100,000 in Dayton, OH (Table 1) and public transit ridership ranged from 0.1% in Pueblo, CO to 57% in New York, NY (Table 2). Fig 1 illustrates the correlation between age-adjusted influenza /pneumonia death rates and public transit ridership in 2015. We ran the adjusted negative binomial model with only the age (and flu year) fixed effects and output the predicted counts, which then were converted to rates using the population estimates. The correlation is negative (-0.525, p<0.001).

**Table 1:** The top and bottom 10 cities by influenza/pneumonia rates for 2015.

	Top 10 Cities	Flu/Pneumonia Deaths per 100,000	Total Population	Bottom 10 Cities	Flu/Pneumonia Deaths per 100,000	Total Population
1	Dayton, OH	329.64	141368	New York, NY	23.44	8426743
2	Birmingham, AL	325.62	212211	Newark, NJ	23.23	279793
3	Pueblo, CO	292.38	30440	Lynn, MA	21.83	91626
4	Salt Lake, UT	291.59	190679	New Orleans, LA	18.58	376738
5	Peoria, IL	282.27	115847	El Paso, TX	18.19	676325
6	Knoxville, TN	278.04	183066	Seattle, WA	10.57	653017
7	Little Rock, AK	270.13	196943	Denver, CO	10.47	649654
8	Tacoma, WA	261.94	203481	Trenton, NJ	7.09	84632
9	Worcester, MA	245.93	183382	Somerville, MA	3.82	78595
10	South Bend, IN	227.66	100590	Springfield, MA	0.65	153947

Source: CDC Weekly Influenza/Pneumonia Deaths from 2015 linked with ACS transit data for 2010–2015 by city, which provides population denominators for rates

**Table 2:** The top and bottom 10 cities by percent transit commuters in 2015.

	Top 10 Cities	Percent individuals over 16 years old who take public transit to work	Bottom 10 Cities	Percent individuals over 16 years old who take public transit to work
1	New York, NY	56.50	Lincoln, NE	1.40
2	Jersey City, NJ	47.62	Little Rock, AR	1.16
3	Washington, DC	37.41	Tulsa, OK	1.08
4	Boston, MA	33.67	Fort Wayne, IN	1.04
5	San Francisco, CA	33.14	Colorado Springs, CO	1.02
6	Somerville, MA	32.31	Montgomery, AL	0.85
7	Cambridge, MA	28.62	Mobile, AL	0.82
8	Chicago, IL	27.63	Boise, ID	0.76
9	Newark, NJ	26.74	Wichita, KS	0.66
10	Yonkers, NY	26.39	Pueblo, CO	0.10

Source: American Community Survey data from 2010–2015 linked with 119 cities from the CDC Weekly Influenza/Pneumonia Deaths from 2015

Note: 2 cities used elsewhere in the analysis do not appear in the 2015 data.

In the baseline negative binomial model, the death rate was negatively associated with percent transit (Table 3). For every percent increase in city-level transit ridership, the rate ratio of influenza/pneumonia deaths, adjusted for age composition, decreased by a factor of 0.98 (IRR = 0.980, B = -0.020, SE = 0.006, p-value = 0.0006). Once we added the other covariates (column B), the relationship remained negative, but was no longer significant (IRR = 0.986, B = -0.014, SE = 0.007, p-value = 0.0559).

**Table 3:** The association between public transit use and influenza/pneumonia deaths in 121 cities across the United States between 2006–2015.

	Model A	Model B
Model	Baseline Negative Binomial (N = 1201)	Adjusted Negative Binomial (N = 1201)
IRR for transit use measure (standard error)	0.980*** (0.968, 0.992)	0.986* (0.972, 1.000)
Model Variables	Includes flu year and age distribution	Also includes region, percent bachelor’s degree or higher, nonwhite, male, below poverty, and unemployed

Note: All standard errors are clustered by city

*p-value < 0.1

**p-value < 0.05

***p-value < 0.01

Table 4 reports results for the various sensitivity analyses. In models using Poisson regression, including flu vaccination as a covariate, limiting the sample to cities where >5% use transit, using commute time over 60 minutes as the primary exposure, and using the ratio of fall/winter influenza/pneumonia mortality to spring/summer mortality (columns A-E), the IRR for the relationship between transit use and influenza/pneumonia mortality remains negative. In the analysis including population density, the IRR for population density (see S1 Appendix) is significantly negative (IRR 0.999, p<0.001), but the IRR for transit use is positive and non-significant (IRR 1.01). Likewise, in the regression including city-specific fixed effects (column H), the IRR for transit use is positive (1.01) but not statistically significant.

**Table 4:** Sensitivity tests on the association between public transit use and influenza/pneumonia deaths in 121 cities across the United States between 2006–2015.

	Model A	Model B	Model C	Model D	Model E	Model F	Model G	Model H
Model	Adjusted Poisson (N = 1201)	Adjusted Negative Binomial including flu vaccination for 2015 (N = 330)	Adjusted Negative Binomial limited to cities with >5% using transit (N = 520)	Adjusted Negative Binomial with exposure as the percent commuting >60 minutes (N = 1201)	Adjusted Negative Binomial adjusting for total death count (N = 1201)	Adjusted Linear Regression with outcome as ratio of flu to pneumonia deaths (N = 963)	Adjusted Negative Binomial including population density (N = 1201)	Fixed Effects Regression (N = 1201)
IRR on % commuting (standard error)	0.984*** (0.972, 0.996)	0.990 (0.972, 1.008)	0.982** (0.964, 1.000)	0.993*** (0.985, 1.001)	0.980*** (0.962, 0.998)	-0.0004 (-0.002, 0.001)	1.013 (0.993, 1.033)	1.01 (0.986, 1.034)
Additional Variables	Same as adjusted negative binomial	Same + city flu vaccination prevalence among 65+	Same as adjusted negative binomial	Same as adjusted negative binomial	Same + total deaths by city/flu year	Same as adjusted negative binomial	Same as adjusted negative binomial adding population density	Fixed effects for cities

Note: All standard errors are clustered by city

*p-value < 0.1

**p-value < 0.05

***p-value < 0.01

**Fig 2:**
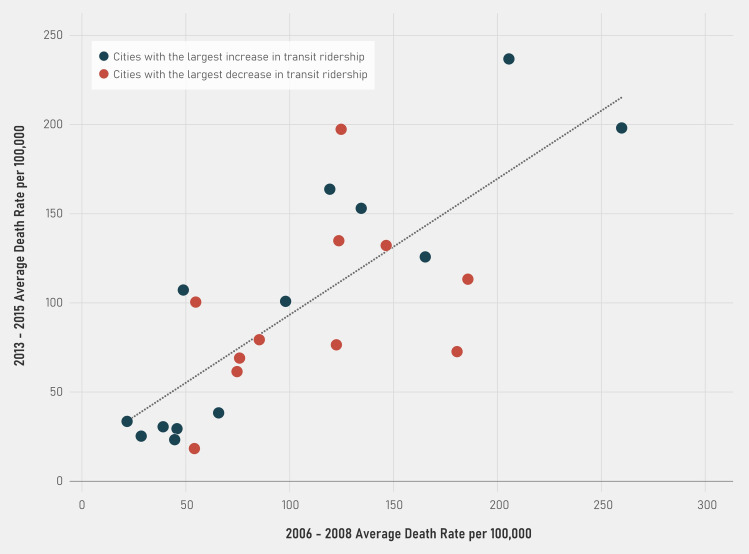
The association between average death rates in 2006–2008 and 2013–2015 in cities with the largest absolute increase and decrease in public transit ridership (n = 24). Source: CDC Weekly Influenza/Pneumonia Deaths from 2006–2016 linked with ACS transit data by city and year for 24 cities with the largest increase and decrease in transit between 2006–2010 and 2011–2015.

When we looked at the 10% of cities with the largest increase in transit use (n = 12; average increase in transit use = 0.02) and the 10% of cities with the largest decrease in transit use (n = 12; average decrease in transit use = -0.02) between the two time periods, death rates in the early period appear highly correlated with death rates in the later period for both sets of cities (i.e., they are clustered fairly symmetrically around the line regardless of whether transit increased or decreased) (Fig 2). The correlation between the change in death rates in the early and late period was 0.99 for those cities with the largest decrease and 0.997 for those with the increase.

**Fig 3:**
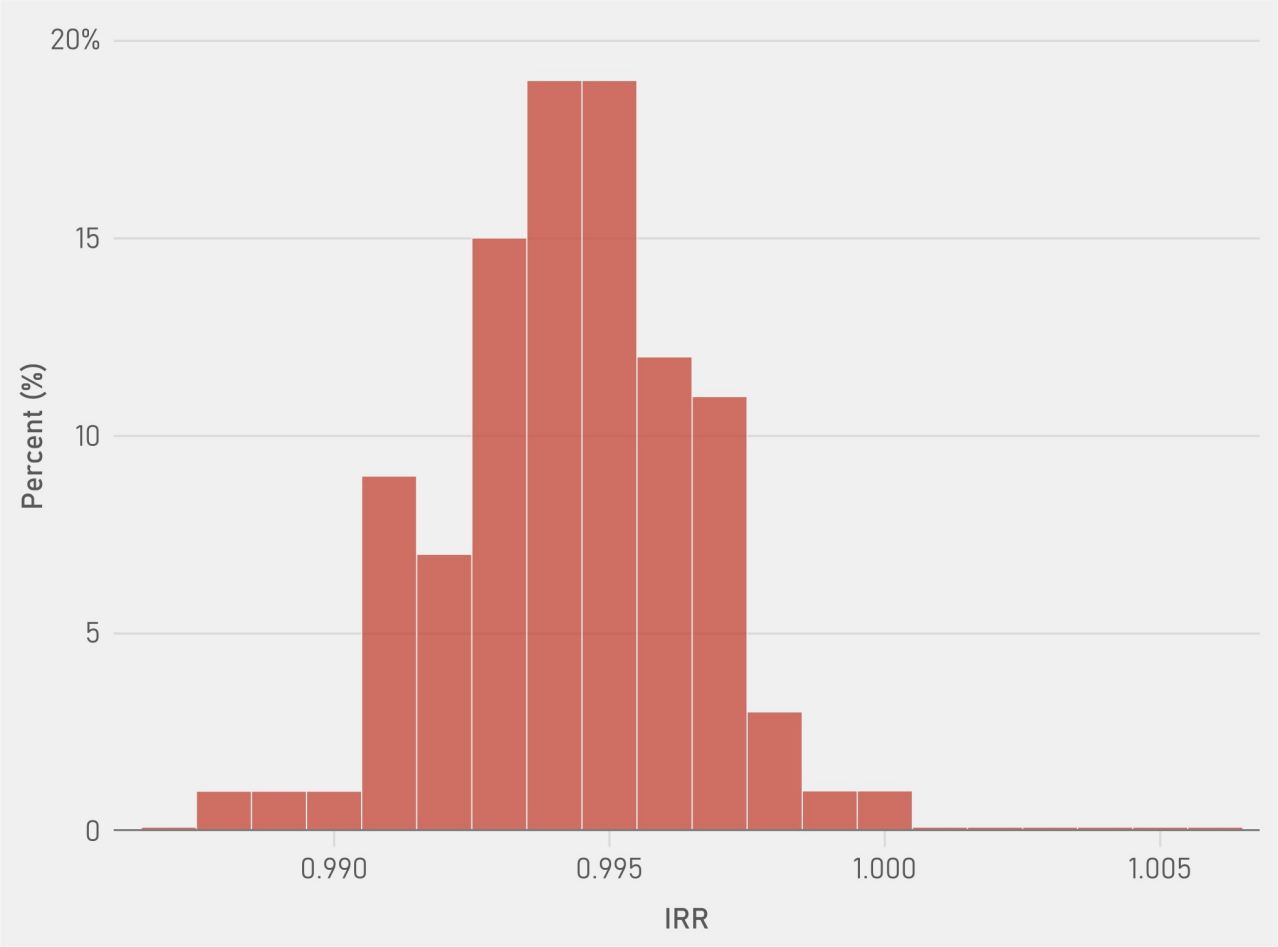
The incidence rate ratio of percent public transit on flu/pneumonia deaths over 100 simulations of the negative binomial model sampling 75% of observations. Source: CDC Weekly Influenza/Pneumonia Deaths from 2006–2016 linked with ACS transit data by city and year, Incidence Rate Ratio from negative binomial model adjusting for flu year, age distribution, region, percent bachelor’s degree or higher, nonwhite, male, below poverty, and unemployed.

In 100 simulations of the data using the adjusted negative binomial model, the IRR ranged from 0.988 to 1.000, with a mean of 0.994. Approximately 99% of all IRR were below 1, implying that the risk of death was reduced the majority of the time (Fig 3).

## Conclusions

Using historical influenza/pneumonia and transit data in the US, we did not find any evidence supporting the hypothesis that greater use of public transit at the city level is associated with local influenza and pneumonia mortality rates. These findings are consistent with prior studies of the relationship between transit use and influenza [8–10], but they do go against convention wisdom. In several of the cities under consideration, over 1 million riders are taken on public transit each day, and riders encounter hundreds of other riders, often in closed, indoor spaces. On the other hand, transit riders generally do not speak to one another and often try to avoid physical contact. More research is needed on the factors that contribute to transmission of viruses in this context.

This study has several limitations. First, we merged datasets from various sources based on city names, however, the geographic boundaries of these cities could be defined differently, meaning the percent of influenza/pneumonia deaths and other covariates could be under or overestimated. Second, this is an ecological study with outcomes and predictors measured at the city-level; therefore, findings should not be interpreted in terms of individual risk. Third, commuting to work is only one aspect of how public transportation is used in a city; there could be meaningful differences in other types of use (e.g., by children, tourists, etc.) that vary between cities and could influence influenza/pneumonia rates. Fourth, we could not separate influenza from pneumonia deaths, many of which are driven by hospital/healthcare acquired infections, not community transmission, creating noise and potentially bias if these infections are not equally spread within the population. That being said, we found good concordance between the CDC’s assessment of flu season severity [16] and our proxy of the ratio of influenza to pneumonia cases, suggesting we are capturing differences in flu severity and not just pneumonia. Specifically, the average ratio of influenza to pneumonia in years that the CDC identified as low flu was 1.20, the average in years that the CDC identified as moderate was 1.25, the average in years that the CDC identified as high was 1.36. There could be unmeasured confounders at the city-level that we did not capture here. Finally, influenza deaths are an imperfect proxy for the prevalence of influenza overall. Influenza mortality conditional on incidence may be confounded by many factors. While we have conducted sensitivity analyses that address some of these (such as controlling for overall death rates and comparing influenza/pneumonia mortality during and outside flu season), other confounders likely remain. In particular, some influenza deaths may not be categorized as either influenza or pneumonia [15].

In conclusion, our research does not address the question of whether, within a city, those who use public transit are at greater risk than those who do not. However, despite widespread concerns about the role of public transit in diffusing respiratory disease, our findings suggest that the rate of utilization is not a singularly important factor in the local prevalence of influenza.

## Supporting information

S1 Appendix (DOCX)

S1 File (XLSX)
