# Supplementary material for: Public transportation and transmission of viral respiratory disease: Evidence from influenza deaths in 121 cities in the United States
Source: PLoS One. 2020 Dec 1;15(12):e0242990. doi: 10.1371/journal.pone.0242990 (PMC7707463; doi:10.1371/journal.pone.0242990)
Supplement: S1 Appendix — (DOCX) [file pone.0242990.s001.docx]

S1 Appendix

Table 1. Sensitivity tests on the association between public transit use and influenza/pneumonia deaths in 121 cities across the United States between 2006-2015, Coefficients and standard error

|  | Model A | Model B | Model C | Model D | Model E | Model F | Model G | Model H | Model I | Model J |
| --- | --- | --- | --- | --- | --- | --- | --- | --- | --- | --- |
| Model | Baseline Negative Binomial (N=1201) | Adjusted Negative Binomial (N=1201) | Adjusted Poisson (N=1201) | Adjusted Negative Binomial including flu vaccination for 2015  (N=330) | Adjusted Negative Binomial limited to cities with >5% using transit (N=520) | Adjusted Negative Binomial with exposure as the percent commuting >60 minutes (N=1201) | Adjusted Negative Binomial adjusting for total death count (N=1201) | Adjusted Linear Regression with outcome as ratio of flu to pneumonia deaths (N=963) | Adjusted Negative Binomial including population density | City-Fixed Effects Regression |
| Coefficient  on % commuting and standard error | -0.0198 (0.0052)*** | -0.0138 (0.0072)* | -0.0164 (0.0062)*** | -0.01  (0.0093) | -0.0177 (0.0089)** | -0.0066 (0.0041) | -0.0204 (0.009)** | -0.0004 (0.0007) | 0.0124 (0.0102) | 0.0092 (0.0122) |
| Additional  Variables | Includes flu year and age distribution | Region, percent bachelor’s degree or higher, nonwhite, male, below poverty, and unemployed | Same as adjusted negative binomial | Same + city flu vaccination prevalence among 65+ | Same as adjusted negative binomial | Same as adjusted negative binomial | Same + total deaths by city/flu year | Same as adjusted negative binomial | Same as adjusted negative binomial adding population density | Fixed effects for cities |

Note: All standard errors are clustered by city

*p-value < 0.1

**p-value < 0.05

***p-value < 0.01

Table 2. Baseline Negative Binomial (N=1201)

| Variables | Coefficient | SE | P-value |
| --- | --- | --- | --- |
| Intercept | -4.3291 | 0.7281 | <.0001 |
| % commuting | -0.0198 | 0.0052 | 0.0006 |
| Flu year 2006 | 0.0079 | 0.0359 | 0.8572 |
| Flu year 2007 | 0.0668 | 0.0359 | 0.1197 |
| Flu year 2008 | 0.0162 | 0.0359 | 0.6876 |
| Flu year 2009 | 0.0465 | 0.0359 | 0.2001 |
| Flu year 2010 | 0.0829 | 0.0359 | 0.0218 |
| Flu year 2011 | 0.0066 | 0.0345 | 0.8291 |
| Flu year 2012 | 0.0381 | 0.0344 | 0.1487 |
| Flu year 2013 | -0.0127 | 0.0345 | 0.5922 |
| Flu year 2014 | 0.0662 | 0.0346 | <.0001 |
| Flu year 2015 | 0 | 0 | . |
| % under 5 yrs | -0.0806 | 0.0324 | 0.0403 |
| % 25-44 yrs | -0.0359 | 0.0115 | 0.0385 |
| % 45-64 yrs | -0.0458 | 0.0154 | 0.0508 |
| % 65+ yrs | 0.0214 | 0.0174 | 0.3617 |

Table 3. Adjusted Negative Binomial (N=1201)

| Variables | Coefficient | SE | P-value |
| --- | --- | --- | --- |
| Intercept | -5.0312 | 2.4654 | 0.0413 |
| % commuting | -0.0138 | 0.0072 | 0.0559 |
| Flu year 2006 | -0.0169 | 0.0823 | 0.8369 |
| Flu year 2007 | 0.0406 | 0.0811 | 0.6165 |
| Flu year 2008 | -0.0079 | 0.0772 | 0.9187 |
| Flu year 2009 | 0.0204 | 0.0728 | 0.7793 |
| Flu year 2010 | 0.0503 | 0.0802 | 0.5307 |
| Flu year 2011 | -0.0196 | 0.0394 | 0.6176 |
| Flu year 2012 | 0.0167 | 0.0331 | 0.6147 |
| Flu year 2013 | -0.0279 | 0.0319 | 0.382 |
| Flu year 2014 | 0.0577 | 0.0186 | 0.002 |
| Flu year 2015 | 0 | 0 | . |
| Region 1 | -0.0504 | 0.2035 | 0.8043 |
| Region 2 | -0.2469 | 0.1868 | 0.1862 |
| Region 4 | -0.2086 | 0.1564 | 0.1823 |
| Region 5 | -0.2634 | 0.1556 | 0.0906 |
| Region 6 | 0.2619 | 0.2364 | 0.2678 |
| Region 7 | -0.5292 | 0.1731 | 0.0022 |
| Region 8 | -0.1896 | 0.2311 | 0.412 |
| Region 9 | 0.0148 | 0.1545 | 0.9235 |
| Region 3 | 0 | 0 | . |
| % under 5 yrs | -0.0998 | 0.053 | 0.0599 |
| % 25-44 yrs | -0.013 | 0.0183 | 0.4778 |
| % 45-64 yrs | -0.0457 | 0.0268 | 0.0885 |
| % 65+ yrs | 0.0198 | 0.0247 | 0.4232 |
| % Bachelor and higher | -0.0151 | 0.0063 | 0.0168 |
| % non-White | -0.0054 | 0.0031 | 0.0819 |
| % male | 0.0191 | 0.0347 | 0.5832 |
| % below poverty | 0.0196 | 0.0224 | 0.3811 |
| % unemployed | -0.0167 | 0.0175 | 0.3393 |

Table 4. Adjusted Poisson (N=1201)

| Variables | Coefficient | SE | P-value |
| --- | --- | --- | --- |
| Intercept | -7.9581 | 2.4954 | 0.0014 |
| % commuting | -0.0164 | 0.0062 | 0.0081 |
| Flu year 2006 | 0.0774 | 0.0643 | 0.229 |
| Flu year 2007 | 0.1334 | 0.0643 | 0.0381 |
| Flu year 2008 | 0.0828 | 0.0609 | 0.1738 |
| Flu year 2009 | 0.1049 | 0.0587 | 0.074 |
| Flu year 2010 | 0.1353 | 0.0565 | 0.0167 |
| Flu year 2011 | -0.0038 | 0.0291 | 0.8955 |
| Flu year 2012 | 0.0365 | 0.0279 | 0.1903 |
| Flu year 2013 | -0.0003 | 0.0208 | 0.9866 |
| Flu year 2014 | 0.0436 | 0.0164 | 0.0081 |
| Flu year 2015 | 0 | 0 | . |
| Region 1 | 0.3474 | 0.1885 | 0.0652 |
| Region 2 | -0.0534 | 0.2218 | 0.8096 |
| Region 4 | 0.0092 | 0.1502 | 0.9511 |
| Region 5 | -0.0945 | 0.1853 | 0.61 |
| Region 6 | 0.3648 | 0.1855 | 0.0492 |
| Region 7 | -0.5477 | 0.1369 | <.0001 |
| Region 8 | -0.2249 | 0.2748 | 0.413 |
| Region 9 | -0.0505 | 0.2397 | 0.8331 |
| Region 3 | 0 | 0 | . |
| % under 5 yrs | -0.0309 | 0.0549 | 0.5729 |
| % 25-44 yrs | -0.0148 | 0.0227 | 0.5145 |
| % 45-64 yrs | -0.0301 | 0.0309 | 0.3293 |
| % 65+ yrs | 0.0522 | 0.0317 | 0.0993 |
| % Bachelor and higher | -0.015 | 0.0075 | 0.0446 |
| % non-White | -0.0042 | 0.0031 | 0.1659 |
| % male | 0.0462 | 0.0425 | 0.277 |
| % below poverty | 0.0386 | 0.0175 | 0.0277 |
| % unemployed | -0.0227 | 0.0174 | 0.1932 |

Table 5. Adjusted Negative Binomial including flu vaccination for 2015 (N=330)

| Variables | Coefficient | SE | P-value |
| --- | --- | --- | --- |
| Intercept | -19.6708 | 8.1813 | 0.0162 |
| % commuting | -0.01 | 0.0093 | 0.2797 |
| Flu year 2011 | -0.0338 | 0.0589 | 0.5665 |
| Flu year 2012 | 0.0198 | 0.0503 | 0.6941 |
| Flu year 2013 | -0.0281 | 0.0471 | 0.5503 |
| Flu year 2014 | 0.0539 | 0.0243 | 0.0268 |
| Flu year 2015 | 0 | 0 | . |
| Region 1 | 0.8055 | 0.4378 | 0.0658 |
| Region 2 | 0.0191 | 0.3174 | 0.9521 |
| Region 4 | -0.6292 | 0.3288 | 0.0556 |
| Region 5 | 0.2637 | 0.2487 | 0.2889 |
| Region 6 | 0.6666 | 0.2702 | 0.0136 |
| Region 7 | -0.2061 | 0.4033 | 0.6092 |
| Region 8 | -0.1782 | 0.34 | 0.6001 |
| Region 9 | 0.0039 | 0.2957 | 0.9896 |
| Region 3 | 0 | 0 | . |
| % under 5 yrs | 0.2852 | 0.2199 | 0.1946 |
| % 25-44 yrs | -0.0824 | 0.045 | 0.0675 |
| % 45-64 yrs | 0.0098 | 0.0605 | 0.8714 |
| % 65+ yrs | 0.129 | 0.0707 | 0.068 |
| % Bachelor and higher | 0.0373 | 0.0247 | 0.1313 |
| % non-White | -0.0117 | 0.0047 | 0.0139 |
| % male | 0.1522 | 0.1198 | 0.2041 |
| % below poverty | 0.0941 | 0.0551 | 0.0874 |
| % unemployed | 0.0669 | 0.0484 | 0.1671 |
| Data_value | 0.0291 | 0.0133 | 0.0292 |

Table 6. Adjusted Negative Binomial limited to cities with >5% using transit (N=520)

| Variables | Coefficient | SE | P-value |
| --- | --- | --- | --- |
| Intercept | -9.2682 | 2.6224 | 0.0004 |
| % commuting | -0.0177 | 0.0089 | 0.0462 |
| Flu year 2006 | 0.1375 | 0.0841 | 0.1021 |
| Flu year 2007 | 0.1953 | 0.0833 | 0.019 |
| Flu year 2008 | 0.1403 | 0.0795 | 0.0775 |
| Flu year 2009 | 0.1004 | 0.0774 | 0.1943 |
| Flu year 2010 | 0.1492 | 0.0736 | 0.0426 |
| Flu year 2011 | 0.0623 | 0.0492 | 0.2053 |
| Flu year 2012 | 0.0937 | 0.0376 | 0.0126 |
| Flu year 2013 | 0.0281 | 0.0335 | 0.4021 |
| Flu year 2014 | 0.0869 | 0.0257 | 0.0007 |
| Flu year 2015 | 0 | 0 | . |
| Region 1 | 0.2665 | 0.3279 | 0.4164 |
| Region 2 | -0.0392 | 0.2874 | 0.8916 |
| Region 4 | -0.3532 | 0.2914 | 0.2254 |
| Region 5 | 0.2257 | 0.2743 | 0.4106 |
| Region 7 | -1.4442 | 0.2952 | <.0001 |
| Region 8 | 0.0241 | 0.5464 | 0.9649 |
| Region 9 | -0.0151 | 0.3125 | 0.9616 |
| Region 3 | 0 | 0 | . |
| % under 5 yrs | -0.0587 | 0.0909 | 0.5181 |
| % 25-44 yrs | -0.0231 | 0.0296 | 0.4364 |
| % 45-64 yrs | -0.0413 | 0.0415 | 0.3189 |
| % 65+ yrs | 0.052 | 0.0409 | 0.2028 |
| % Bachelor and higher | -0.013 | 0.0096 | 0.1756 |
| % non-White | -0.0137 | 0.0049 | 0.0054 |
| % male | 0.0974 | 0.0653 | 0.1357 |
| % below poverty | 0.0312 | 0.025 | 0.2125 |
| % unemployed | -0.0077 | 0.023 | 0.7371 |

Table 7. Adjusted Negative Binomial with exposure as the percent commuting >60 minutes (N=1201)

| Intercept | -5.0716 | 2.4805 | 0.0409 |
| --- | --- | --- | --- |
| % commuting60PLUSMIN | -0.0066 | 0.0041 | 0.1013 |
| Flu year 2006 | -0.0576 | 0.0853 | 0.4998 |
| Flu year 2007 | -0.0017 | 0.084 | 0.9835 |
| Flu year 2008 | -0.0503 | 0.0798 | 0.5282 |
| Flu year 2009 | -0.0193 | 0.0761 | 0.8002 |
| Flu year 2010 | 0.0073 | 0.0847 | 0.9317 |
| Flu year 2011 | -0.0222 | 0.0394 | 0.5728 |
| Flu year 2012 | 0.0127 | 0.0334 | 0.7039 |
| Flu year 2013 | -0.0281 | 0.0314 | 0.3706 |
| Flu year 2014 | 0.0586 | 0.0185 | 0.0016 |
| Flu year 2015 | 0 | 0 | . |
| Region 1 | -0.0518 | 0.2042 | 0.7998 |
| Region 2 | -0.3589 | 0.1677 | 0.0323 |
| Region 4 | -0.2062 | 0.1579 | 0.1916 |
| Region 5 | -0.1975 | 0.1548 | 0.202 |
| Region 6 | 0.3761 | 0.245 | 0.1248 |
| Region 7 | -0.3637 | 0.1863 | 0.0509 |
| Region 8 | -0.0955 | 0.2368 | 0.6868 |
| Region 9 | 0.1415 | 0.1655 | 0.3923 |
| Region 3 | 0 | 0 | . |
| % under 5 yrs | -0.0867 | 0.0496 | 0.0803 |
| % 25-44 yrs | -0.0191 | 0.0166 | 0.2501 |
| % 45-64 yrs | -0.0385 | 0.0283 | 0.173 |
| % 65+ yrs | 0.0123 | 0.0259 | 0.6351 |
| % Bachelor and higher | -0.022 | 0.0057 | 0.0001 |
| % non-White | -0.0073 | 0.0027 | 0.0067 |
| % male | 0.0268 | 0.0348 | 0.4406 |
| % below poverty | 0.0214 | 0.0218 | 0.3261 |
| % unemployed | -0.017 | 0.0182 | 0.3523 |

Table 8. Adjusted Negative Binomial adjusting for total death count (N=1201)

| Variables | Coefficient | SE | P-value |
| --- | --- | --- | --- |
| Intercept | -6.3202 | 2.791 | 0.0235 |
| % commuting | -0.0204 | 0.009 | 0.0236 |
| Flu year 2006 | 0.0031 | 0.0898 | 0.9729 |
| Flu year 2007 | 0.0678 | 0.0884 | 0.4428 |
| Flu year 2008 | 0.0181 | 0.085 | 0.8318 |
| Flu year 2009 | 0.0519 | 0.0803 | 0.5179 |
| Flu year 2010 | 0.0812 | 0.0879 | 0.3558 |
| Flu year 2011 | 0.0135 | 0.0382 | 0.7242 |
| Flu year 2012 | 0.0403 | 0.0315 | 0.2007 |
| Flu year 2013 | -0.0103 | 0.0325 | 0.7522 |
| Flu year 2014 | 0.0627 | 0.0179 | 0.0005 |
| Flu year 2015 | 0 | 0 | . |
| Region 1 | 0.2444 | 0.228 | 0.2837 |
| Region 2 | -0.0613 | 0.1894 | 0.7464 |
| Region 4 | -0.1485 | 0.1705 | 0.3836 |
| Region 5 | -0.1968 | 0.1744 | 0.2591 |
| Region 6 | 0.1367 | 0.2119 | 0.5189 |
| Region 7 | -0.6305 | 0.2005 | 0.0017 |
| Region 8 | -0.3443 | 0.2257 | 0.1272 |
| Region 9 | 0.0429 | 0.1675 | 0.798 |
| Region 3 | 0 | 0 | . |
| % under 5 yrs | -0.0717 | 0.0595 | 0.2283 |
| % 25-44 yrs | -0.0187 | 0.0196 | 0.3409 |
| % 45-64 yrs | -0.048 | 0.028 | 0.0865 |
| % 65+ yrs | 0.0268 | 0.0277 | 0.3323 |
| % Bachelor and higher | -0.0138 | 0.0074 | 0.0623 |
| % non-White | -0.0067 | 0.0034 | 0.0466 |
| % male | 0.0369 | 0.0383 | 0.335 |
| % below poverty | 0.0189 | 0.0245 | 0.4411 |
| % unemployed | -0.0165 | 0.019 | 0.3834 |
| alldeaths | 0.0001 | 0 | <.0001 |

Table 9. Adjusted Linear Regression with outcome as ratio of flu to pneumonia deaths (N=963)

| Variables | Coefficient | SE | P-value |
| --- | --- | --- | --- |
| Intercept | 0.1874 | 0.1878 | 0.3181 |
| % commuting | -0.0004 | 0.0007 | 0.6167 |
| Flu year 2006 | -0.0027 | 0.0051 | 0.6005 |
| Flu year 2007 | 0.004 | 0.0051 | 0.4331 |
| Flu year 2008 | -0.0013 | 0.0051 | 0.8025 |
| Flu year 2009 | 0.0021 | 0.0051 | 0.6791 |
| Flu year 2010 | 0.0056 | 0.0051 | 0.2701 |
| Flu year 2011 | 0.0004 | 0.0037 | 0.9168 |
| Flu year 2012 | 0.0035 | 0.0037 | 0.3396 |
| Flu year 2013 | -0.0019 | 0.0037 | 0.6164 |
| Flu year 2014 | 0.0072 | 0.0037 | 0.0528 |
| Flu year 2015 | 0 | 0 | . |
| Region 1 | -0.0152 | 0.0198 | 0.4419 |
| Region 2 | -0.0315 | 0.0183 | 0.085 |
| Region 4 | -0.0304 | 0.0196 | 0.1206 |
| Region 5 | -0.035 | 0.0185 | 0.059 |
| Region 6 | 0.0386 | 0.0232 | 0.0969 |
| Region 7 | -0.0385 | 0.0174 | 0.0268 |
| Region 8 | -0.0263 | 0.0216 | 0.2228 |
| Region 9 | -0.0053 | 0.0203 | 0.7941 |
| Region 3 | 0 | 0 | . |
| % under 5 yrs | -0.0116 | 0.0039 | 0.003 |
| % 25-44 yrs | -0.0017 | 0.0015 | 0.2584 |
| % 45-64 yrs | -0.0036 | 0.0018 | 0.0437 |
| % 65+ yrs | 0.0002 | 0.0022 | 0.9311 |
| % Bachelor and higher | -0.0019 | 0.0007 | 0.0052 |
| % non-White | -0.0005 | 0.0003 | 0.1053 |
| % male | 0.0048 | 0.003 | 0.1051 |
| % below poverty | 0.0022 | 0.0013 | 0.0852 |
| % unemployed | -0.0027 | 0.0012 | 0.0268 |

Table 10. Adjusted Negative Binomial including population density

| Variables | Coefficient | SE | P-value |
| --- | --- | --- | --- |
| Intercept | -6.4453 | 2.5861 | 0.0127 |
| % commuting | 0.0124 | 0.0102 | 0.2211 |
| Flu year 2006 | -0.0076 | 0.0854 | 0.9287 |
| Flu year 2007 | 0.0515 | 0.0843 | 0.5419 |
| Flu year 2008 | 0.0018 | 0.0809 | 0.9823 |
| Flu year 2009 | 0.0272 | 0.0769 | 0.7237 |
| Flu year 2010 | 0.0584 | 0.0851 | 0.4921 |
| Flu year 2011 | -0.0147 | 0.0391 | 0.707 |
| Flu year 2012 | 0.0193 | 0.0331 | 0.5606 |
| Flu year 2013 | -0.0227 | 0.0318 | 0.476 |
| Flu year 2014 | 0.0602 | 0.0181 | 0.0009 |
| Flu year 2015 | 0 | 0 | . |
| Region 1 | 0.1661 | 0.201 | 0.4086 |
| Region 2 | -0.0347 | 0.1748 | 0.8427 |
| Region 4 | -0.3059 | 0.1506 | 0.0423 |
| Region 5 | -0.3256 | 0.1489 | 0.0287 |
| Region 6 | 0.1026 | 0.2304 | 0.6559 |
| Region 7 | -0.6962 | 0.1839 | 0.0002 |
| Region 8 | -0.3193 | 0.2216 | 0.1495 |
| Region 9 | 0.0939 | 0.1631 | 0.5648 |
| Region 3 | 0 | 0 | . |
| % under 5 yrs | -0.0819 | 0.0499 | 0.1009 |
| % 25-44 yrs | -0.0101 | 0.0173 | 0.5605 |
| % 45-64 yrs | -0.0417 | 0.0275 | 0.1292 |
| % 65+ yrs | 0.027 | 0.0266 | 0.3104 |
| % Bachelor and higher | -0.0127 | 0.0071 | 0.0739 |
| % non-White | -0.0017 | 0.0035 | 0.6256 |
| % male | 0.0428 | 0.0343 | 0.2124 |
| % below poverty | 0.0174 | 0.0222 | 0.4326 |
| % unemployed | -0.0245 | 0.0169 | 0.1479 |
| pop_density | -0.0001 | 0 | <.0001 |

Table 11. City-Fixed Effects Regression

| Variables | Category | DF | Coefficient | SE | P-value |
| --- | --- | --- | --- | --- | --- |
| Intercept |  | 1 | -7.6568 | 2.0912 | 0.0003 |
| % commuting |  | 1 | 0.0092 | 0.0122 | 0.4513 |
| Flu year 2006 |  | 1 | 0.0063 | 0.0535 | 0.9069 |
| Flu year 2007 |  | 1 | 0.0639 | 0.0534 | 0.2314 |
| Flu year 2008 |  | 1 | -0.0038 | 0.0535 | 0.9436 |
| Flu year 2009 |  | 1 | 0.0203 | 0.0537 | 0.7054 |
| Flu year 2010 |  | 1 | 0.0585 | 0.0536 | 0.2753 |
| Flu year 2011 |  | 1 | 0.01 | 0.0324 | 0.7584 |
| Flu year 2012 |  | 1 | 0.0356 | 0.0322 | 0.2689 |
| Flu year 2013 |  | 1 | 0.0134 | 0.0323 | 0.6789 |
| Flu year 2014 |  | 1 | 0.0768 | 0.0323 | 0.0173 |
| Flu year 2015 |  | 0 | 0 | 0 | . |
| % under 5 yrs |  | 1 | -0.0535 | 0.0389 | 0.1696 |
| % 25-44 yrs |  | 1 | 0.0093 | 0.0156 | 0.5531 |
| % 45-64 yrs |  | 1 | -0.0274 | 0.0173 | 0.1144 |
| % 65+ yrs |  | 1 | 0.028 | 0.0232 | 0.227 |
| % Bachelor and higher |  | 1 | -0.0356 | 0.0094 | 0.0001 |
| % non-White |  | 1 | -0.0181 | 0.0052 | 0.0005 |
| % male |  | 1 | 0.0692 | 0.0312 | 0.0264 |
| % below poverty |  | 1 | 0.0336 | 0.0129 | 0.0089 |
| % unemployed |  | 1 | -0.0406 | 0.0116 | 0.0005 |
| City | Akron | 1 | -0.8053 | 0.1694 | <.0001 |
| City | Albany | 1 | -0.1161 | 0.239 | 0.6271 |
| City | Albuqu | 1 | -0.1983 | 0.243 | 0.4145 |
| City | Allent | 1 | -1.1334 | 0.1837 | <.0001 |
| City | Atlant | 1 | 0.1147 | 0.2847 | 0.687 |
| City | Austin | 1 | -0.9535 | 0.2742 | 0.0005 |
| City | Baltim | 1 | 0.1655 | 0.2895 | 0.5675 |
| City | Baton | 1 | -1.186 | 0.2468 | <.0001 |
| City | Berkel | 1 | -0.1933 | 0.4446 | 0.6636 |
| City | Birmin | 1 | 1.4218 | 0.2558 | <.0001 |
| City | Boise | 1 | -0.8154 | 0.2138 | 0.0001 |
| City | Boston | 1 | -0.1725 | 0.3876 | 0.6562 |
| City | Bridge | 1 | -0.05 | 0.2459 | 0.839 |
| City | Buffal | 1 | -0.3734 | 0.1924 | 0.0523 |
| City | Cambri | 1 | 0.1334 | 0.4477 | 0.7658 |
| City | Camden | 1 | -0.092 | 0.2696 | 0.7331 |
| City | Canton | 1 | -0.1613 | 0.2176 | 0.4586 |
| City | Charlo | 1 | -0.1651 | 0.2738 | 0.5467 |
| City | Chatta | 1 | 0.0426 | 0.1751 | 0.808 |
| City | Chicag | 1 | -1.1503 | 0.3265 | 0.0004 |
| City | Cincin | 1 | -0.0612 | 0.1903 | 0.7479 |
| City | Clevel | 1 | 0.1458 | 0.1769 | 0.4097 |
| City | Colora | 1 | -1.4472 | 0.2188 | <.0001 |
| City | Columb | 1 | -0.4453 | 0.2162 | 0.0394 |
| City | Corpus | 1 | -0.4552 | 0.23 | 0.0479 |
| City | Dallas | 1 | -0.7335 | 0.2432 | 0.0026 |
| City | Dayton | 1 | 0.4583 | 0.1547 | 0.003 |
| City | Denver | 1 | -1.2253 | 0.2376 | <.0001 |
| City | Des Mo | 1 | -0.5395 | 0.2176 | 0.0132 |
| City | Detroi | 1 | -0.1129 | 0.2551 | 0.658 |
| City | Duluth | 1 | -0.8322 | 0.2103 | <.0001 |
| City | El Pas | 1 | -1.1727 | 0.3014 | <.0001 |
| City | Elizab | 1 | -0.8295 | 0.2827 | 0.0033 |
| City | Erie | 1 | -0.6986 | 0.1981 | 0.0004 |
| City | Evansv | 1 | -0.8691 | 0.2238 | 0.0001 |
| City | Fall R | 1 | -0.924 | 0.2502 | 0.0002 |
| City | Fort W | 1 | -0.9245 | 0.2046 | <.0001 |
| City | Fresno | 1 | -0.052 | 0.2068 | 0.8015 |
| City | Gary | 1 | -0.9771 | 0.2899 | 0.0007 |
| City | Glenda | 1 | 0.3677 | 0.2463 | 0.1354 |
| City | Grand | 1 | -0.3602 | 0.174 | 0.0385 |
| City | Hartfo | 1 | 0.6736 | 0.2457 | 0.0061 |
| City | Honolu | 1 | 0.2683 | 0.4001 | 0.5025 |
| City | Housto | 1 | -1.0977 | 0.2571 | <.0001 |
| City | Indian | 1 | -0.5059 | 0.2006 | 0.0117 |
| City | Jackso | 1 | -0.9971 | 0.2116 | <.0001 |
| City | Jersey | 1 | -0.847 | 0.5455 | 0.1205 |
| City | Kansas | 1 | -0.8568 | 0.184 | <.0001 |
| City | Knoxvi | 1 | -0.1047 | 0.1889 | 0.5796 |
| City | Lansin | 1 | -0.2951 | 0.1774 | 0.0962 |
| City | Las Ve | 1 | 0.0845 | 0.1885 | 0.6539 |
| City | Lexing | 1 | -1.0907 | 0.2151 | <.0001 |
| City | Lincol | 1 | -1.5184 | 0.2147 | <.0001 |
| City | Little | 1 | 0.2378 | 0.269 | 0.3767 |
| City | Long B | 1 | -0.1193 | 0.2448 | 0.6259 |
| City | Los An | 1 | -0.9978 | 0.2523 | <.0001 |
| City | Lowell | 1 | -0.5171 | 0.21 | 0.0138 |
| City | Lynn | 1 | -1.6677 | 0.2351 | <.0001 |
| City | Memphi | 1 | 0.336 | 0.2382 | 0.1584 |
| City | Miami | 1 | -0.552 | 0.3189 | 0.0835 |
| City | Milwau | 1 | -1.1794 | 0.1922 | <.0001 |
| City | Minnea | 1 | -0.746 | 0.2398 | 0.0019 |
| City | Mobile | 1 | 0.1552 | 0.2125 | 0.4654 |
| City | Montgo | 1 | 0.1255 | 0.2646 | 0.6353 |
| City | Nashvi | 1 | -0.3677 | 0.2254 | 0.1028 |
| City | New Be | 1 | -1.2592 | 0.2197 | <.0001 |
| City | New Ha | 1 | 0.3882 | 0.2612 | 0.1372 |
| City | New Or | 1 | -1.6353 | 0.2751 | <.0001 |
| City | New Yo | 1 | -1.5167 | 0.6405 | 0.0179 |
| City | Newark | 1 | -1.3444 | 0.3213 | <.0001 |
| City | Norfol | 1 | -1.4239 | 0.2052 | <.0001 |
| City | Ogden | 1 | -0.6426 | 0.2103 | 0.0023 |
| City | Omaha | 1 | -0.6114 | 0.2049 | 0.0028 |
| City | Pasade | 1 | 0.3885 | 0.3524 | 0.2704 |
| City | Paters | 1 | -1.156 | 0.3035 | 0.0001 |
| City | Peoria | 1 | 0.4485 | 0.2005 | 0.0253 |
| City | Philad | 1 | -1.3199 | 0.3202 | <.0001 |
| City | Phoeni | 1 | -1.4916 | 0.2076 | <.0001 |
| City | Pittsb | 1 | -0.3215 | 0.2502 | 0.1988 |
| City | Portla | 1 | -0.9592 | 0.2396 | <.0001 |
| City | Provid | 1 | -0.1485 | 0.2298 | 0.5182 |
| City | Pueblo | 1 | -0.6897 | 0.1688 | <.0001 |
| City | Readin | 1 | -0.2255 | 0.1769 | 0.2023 |
| City | Richmo | 1 | -0.3916 | 0.2269 | 0.0843 |
| City | Roches | 1 | 0.3213 | 0.1885 | 0.0884 |
| City | Rockfo | 1 | -0.5637 | 0.1617 | 0.0005 |
| City | Sacram | 1 | 0.7772 | 0.2353 | 0.001 |
| City | Saint | 1 | -0.6275 | 0.1848 | 0.0007 |
| City | Salt L | 1 | 0.3292 | 0.2169 | 0.1292 |
| City | San An | 1 | -0.5363 | 0.2544 | 0.0351 |
| City | San Di | 1 | -0.669 | 0.27 | 0.0132 |
| City | San Fr | 1 | -0.3477 | 0.4609 | 0.4506 |
| City | San Jo | 1 | 0.2443 | 0.3214 | 0.4471 |
| City | Santa | 1 | 0.4447 | 0.3137 | 0.1563 |
| City | Savann | 1 | 0.1191 | 0.2093 | 0.5693 |
| City | Schene | 1 | -0.4625 | 0.2011 | 0.0215 |
| City | Scrant | 1 | -1.2977 | 0.2018 | <.0001 |
| City | Seattl | 1 | -0.8097 | 0.3324 | 0.0149 |
| City | Shreve | 1 | 0.0264 | 0.225 | 0.9065 |
| City | Somerv | 1 | -4.3259 | 0.4873 | <.0001 |
| City | South | 1 | -0.0863 | 0.164 | 0.5988 |
| City | Spokan | 1 | -0.7698 | 0.1982 | 0.0001 |
| City | Spring | 1 | -0.454 | 0.2118 | 0.032 |
| City | Syracu | 1 | 0.1088 | 0.1873 | 0.5613 |
| City | Tacoma | 1 | -0.022 | 0.1895 | 0.9077 |
| City | Tampa | 1 | 0.0428 | 0.2174 | 0.844 |
| City | Toledo | 1 | -0.9537 | 0.1731 | <.0001 |
| City | Trento | 1 | -1.6434 | 0.2459 | <.0001 |
| City | Tucson | 1 | -0.9128 | 0.1697 | <.0001 |
| City | Tulsa | 1 | -0.2231 | 0.1995 | 0.2634 |
| City | Utica | 1 | -1.3087 | 0.1837 | <.0001 |
| City | Washin | 1 | -0.2785 | 0.4698 | 0.5533 |
| City | Waterb | 1 | -0.3698 | 0.2083 | 0.0758 |
| City | Wichit | 1 | -0.9511 | 0.1972 | <.0001 |
| City | Wilmin | 1 | -0.1243 | 0.2537 | 0.6242 |
| City | Worces | 1 | 0.2935 | 0.1988 | 0.1399 |
| City | Yonker | 1 | -1.3568 | 0.3644 | 0.0002 |
| City | Youngs | 0 | 0 | 0 | . |
